# Neglected Elbow Dislocation: A Retrospective Look at Functional Recovery with Posterior Approach Surgery

**DOI:** 10.5704/MOJ.2603.014

**Published:** 2026-03

**Authors:** SA Hadinoto, T Sumarwoto, M Frityatama

**Affiliations:** 1 Orthopaedic and Traumatology Department, Soeharso Orthopaedic Hospital, Surakarta, Indonesia; 2 Faculty of Medicine, Sebelas Maret University, Surakarta, Indonesia

**Keywords:** neglected elbow dislocation, posterior approach, DASH, MEPI

## Abstract

**Introduction:**

Elbow dislocation is the second most frequent joint dislocation after the shoulder. In developing nations, untreated elbow dislocations are common, making management of neglected cases particularly challenging. Treatment approaches depend on the time since injury and the patient’s age. This study evaluates the outcomes of posterior approach surgery for neglected elbow dislocations. **Materials and methods:** This retrospective study reviewed patients with neglected elbow dislocations treated via posterior approach surgery between January 2020 and December 2023 at Soeharso Orthopedic Hospital. Data collected included patient demographics, onset-to-surgery duration, and post-operative outcomes assessed by DASH and MEPI scores. All surgeries were performed by a board-certified hand and upper extremity surgeon. Data were analysed using Microsoft Office and IBM SPSS Statistics.

**Results:**

A total of 23 patients, predominantly male adults, underwent posterior approach surgery. The average time from onset to surgery was 7.83 months. Post-operative outcomes showed satisfactory results, with mean DASH and MEPI scores of 15.32 and 90, respectively.

**Conclusion:**

Neglected elbow dislocations primarily affect adult males. The posterior approach offers several advantages and yields good functional outcomes, as demonstrated by DASH and MEPI scores.

## INTRODUCTION

The elbow plays a crucial role in positioning the hand for a variety of tasks. Elbow flexion, combined with supination, enables tasks such as bringing the hand towards the body and face for eating, dressing, and personal hygiene, as well as for pulling or carrying objects^1^. The most frequent type of joint dislocation in upper extremity is elbow dislocation. It is only surpassed by shoulder dislocations in the adult population. Roughly 20% of all articular dislocations occur at this rate. Elbow dislocations are posterior or posterolateral in at least 90% of cases. When the elbow dislocation presents, it is distorted either in minimum flexion or extension, resulting in a restricted range of motion that makes it challenging to carry out daily tasks^1,2^.

These kinds of presentations are far more prevalent in developing and poor countries. Low socioeconomic position, lack of education, ignorance of the seriousness of the injury, procrastination in seeking treatment, and the fact that most patients first seek out local bonesetters for manipulation and massage, which simply makes the issue worse, are all potential contributing factors^1-3^.

This illness is difficult to manage because of the high rate of complications and unpredictable results. These will cause the elbow to become immobile in an extended position, which will cause the triceps muscle and collateral ligaments to retract. The elbow becomes extremely rigid as a result, making surgery challenging^1,4^.

Depending on the extent of the injury and the condition of the joint, the available treatment options include elbow arthroplasty, open reduction, ligament repair/reconstruction, and an external fixator. A single-step treatment to minimise and repair the neglected dislocated elbow would be optimal, despite its technical difficulty to allow for an early restoration to function and prevent instability^4^. When elbow dislocation is neglected, the elbow is immobile in either extension or flexion, has a limited range of motion for activities of daily living, and only a few degrees of flexion, supination, or pronation. The best course of action is determined by the patient's age and the amount of time that has passed between the injury and therapy^5^.

This study aims to present the profile and functional outcomes of patients with neglected elbow dislocation who underwent surgery using a posterior approach. The research displays patient characteristics and patient outcomes.

## MATERIALS AND METHODS

This research is a retrospective study. This research was conducted to present the profile and functional outcomes of patients with neglected elbow dislocation who underwent surgery with a posterior approach only. The research sample was neglected elbow dislocation patients who underwent surgery with a posterior approach in the period January 2020 - December 2023 at Soeharso Orthopaedic Hospital. All surgeries are performed by one board-certified hand and upper extremity surgeon (SAH). Surgical indications included pain, reduced functional range of motion, and stiffness. Cases involving fractures, conservative treatment, or double posterior approach surgery were excluded. Furthermore, patients with acute dislocations, those lost to follow-up, and those who refused surgery or participation were excluded from the study. Informed consent was obtained from the study participants prior to the commencement of the study.

**Fig. 1:**
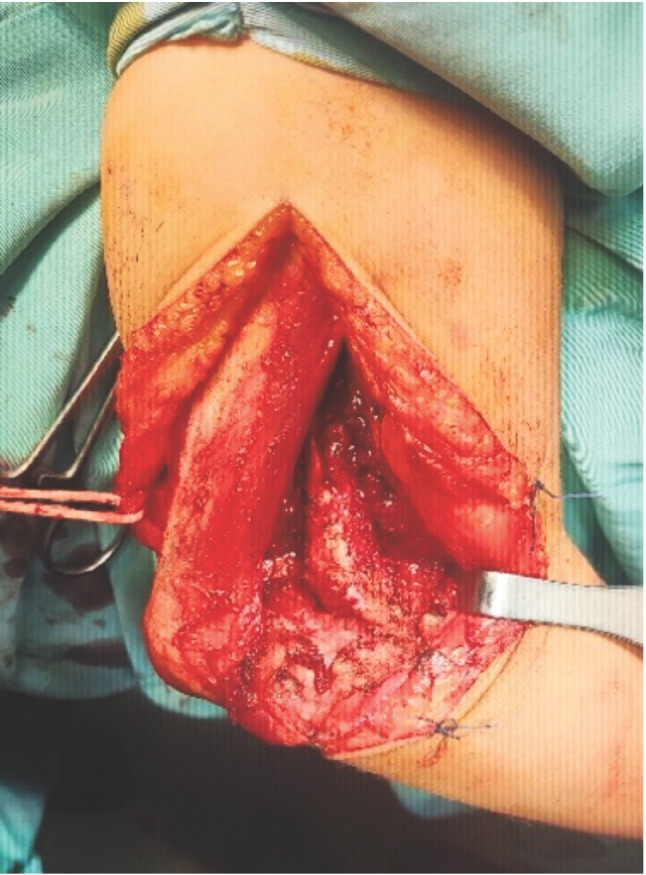
Posterior approach. The ulnar nerve was tagged.

Open reduction under general anaesthesia was performed through the posterior approaches of the elbow (Fig. 1). A tourniquet cuff was placed proximally on the arm and inflated before the incision. Longitudinal incision over posterior aspect of elbow begins 5cm above the olecranon in midline of posterior aspect of arm. At the tip of olecranon it is curved laterally. Distally, it is curved again medially towards middle of ulna. Paratricipital (Allonso Liames) approach is performed. The deep fascia is incised in the midline and ulnar nerve is identified, dissected out, protected and marked with a nerve tape. The ulnar nerve is released distally up to the first branch of the FCU (Flexor Carpi Ulnaris).

**Fig. 2:**
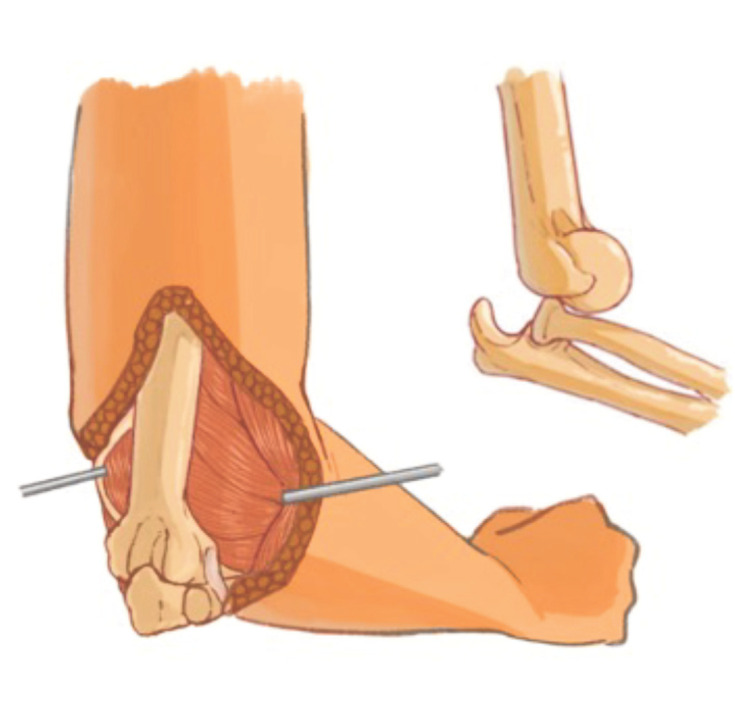
Lateral-medial and posterior capsulotomies were performed. All dense fibrous tissue removed.

Lateral-medial and posterior capsulotomies were performed, the shortened medial and lateral collateral ligaments were released, anterior capsulotomies were performed from the lateral side and the contracted capsules were opened to access the articular surfaces using blunt rasps. Be careful not to injure the capitellum and trochlea articular surfaces. All dense fibrous tissue in the olecranon fossa, coronoid fossa, radial head, distal humerus-proximal radius, and ulna should be carefully removed, followed by circumferential capsular release. Joint surfaces were preserved in all patients. The elbow was then reduced, and reduction of the ulnotrochlear and radiocapitellar joints was achieved by manipulation with slow, gentle, progressive manoeuvres to avoid sudden movements that could damage the cartilage and nerve (Fig. 2).

**Fig. 3:**
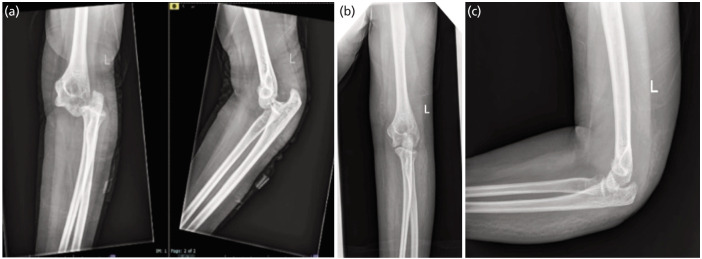
(a) Radiographs of neglected elbow fracture. (b and c) Radiographs after open reduction using posterior approach.

The elbow was flexed maximally to stretch the triceps, sometimes triceps lengthening is performed by multiple stab incisions if there is severe contracture especially if the dislocation is more than three months. Capsule and collateral ligaments are sutured back together via transosseus suture of the footprint or suturing using anchor site after the reduction. Hemostasis was ascertained after the removal of the tourniquet. A 3mm drain was inserted, and the wounds were closed in layers. The posterior above-elbow plaster of Paris was adjusted as a back slab for a period of three–four weeks, following which rehabilitation under the observation of the occupational therapist was initiated (Fig. 3).

**Fig. 4:**
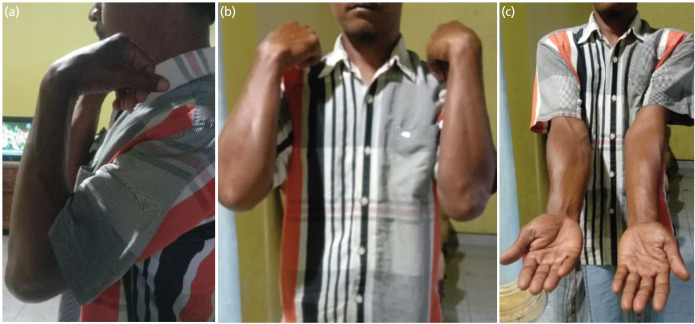
Elbow range of motion after open reduction using posterior approach surgery in patient with good DASH and MEPI score.

Patients were assessed for follow-up for at least six months post-operatively in the outpatient clinic and contacted by phone to ascertain functional recovery (Fig. 4). Ethical approval was obtained from the institutional ethics committee before beginning the study. The data analysed includes age, gender, length of treatment days, onset before surgery and outcome based on DASH (Disabilities of Arm, Shoulder, and Hand) and MEPI (Mayo Elbow Performance Index) scores. The data was then analysed using Microsoft Office Software and IBM SPSS Statistics.

## RESULTS

This study had a research sample of 23 patients with neglected elbow dislocation. The gender dominance of the study was male, up to 65%. The greatest prevalence was found in the adult age group with an average age of 34 years. Patients had different disease onsets until surgery was performed, the average onset was 7.83 months. The average length of treatment for patients is 3.7 days.

Table I shows the characteristics of the research subjects and elbow function outcomes. At least six months after surgery, the patient was followed-up again and assessed for elbow function outcomes with DASH and MEPI scores. The mean DASH score in this study was 15.3 (4.2–39.9) and the mean MEPI score was 90 (60–100). This shows that the elbow function outcomes according to DASH was good and the MEPI results was excellent.

**Table I:** Characteristics of research subject.

**Characteristic**	**Total (%)**
Sex	
Male	15 (65.22)
Female	8 (34.78)
Age	
Children <18 years old	5 (21.74)
Adult 18 – 50 years old	16 (69.57)
Elderly >50 years old	2 (8.69)
Length of treatment days	
<5 days	21 (91.31)
>5 days	2 (8.69)
Onset before surgery	
<3 months	10 (43.48)
3 – 6 months	4 (17.39)
>6 months	9 (39.13)
DASH	
Excellent	5 (21.74)
Good	10 (43.48)
Fair	7 (30.43)
Poor	1 (4.35)
MEPI	
Excellent	12 (52.17)
Good	10 (43.48)
Fair	1 (4.35)
Poor	0 (0)

Table II shows that there is a no statistically significant relationship between gender and patient outcomes. This study has p>0.05. The predominance of research subjects had good results on the DASH score and excellent results on the MEPI score. The correlation coefficient shows that male patients have a lower DASH score and a higher MEPI score. Table III shows a very significant correlation between age and elbow function outcomes according to MEPI score status. The correlation coefficient shows that patients with a younger age have a lower DASH score and a higher MEPI value.

**Table II:** Correlation between sex and outcome.

**Variable**	**Excellent**	**Good**	**DASH** **Fair**	**Poor**	**p**	**R**
Male	5	7	3	0	0.125	0.329
Female	0	3	4	1		
**Variable**	**Excellent**	**Good**	**MEPI** **Fair**	**Poor**	**p**	**R**
Male	9	6	0	0	0.083	-0.369
Female	3	4	1	0		

**Table III:** Correlation between age and outcome.

**Variable**	**Excellent**	**Good**	**DASH** **Fair**	**Poor**	**p**	**R**
Children	3	2	0	0	0.086	0.366
Adult	2	7	7	1		
Elderly	0	1	0	0		
**Variable**	**Excellent**	**Good**	**MEPI** **Fair**	**Poor**	**p**	**R**
Children	5	9	0	0	0.028	-0.458
Adult	7	1	1	0		
Elderly	0	0	0	0		

Table IV shows that there is a no statistically significant relationship between onset before surgery and patient outcomes. The correlation coefficient shows that patient with onset before surgery less than three months have a lower DASH score and a higher MEPI score.

**Table IV:** Correlation between onset before surgery and outcome.

**Variable**	**Excellent**	**Good**	**DASH** **Fair**	**Poor**	**p**	**R**
<3 months	4	4	1	0	0.490	0.152
3 – 6 months	0	3	0	1		
>6 months	1	3	6	0		
**Variable**	**Excellent**	**Good**	**MEPI** **Fair**	**Poor**	**p**	**R**
<3 months	6	3	0	0	0.400	0.184
3 – 6 months	3	0	1	0		
>6 months	3	7	0	0		

## DISCUSSION

The primary objective in treating chronically unreduced elbow dislocation is to achieve a stable, cantered joint and recover an adequate range of motion. Managing chronic elbow dislocation presents a challenge for trauma surgeons as it involves balancing the seemingly contradictory aims of restoring stability while also improving motion range^6,7^. Neglected dislocation of the elbow refers to traumatic dislocation of the elbow joint that has gone untreated for three weeks or more. These are far more prevalent in undeveloped and developing countries. The patient is unable to execute activities of daily living due to a painful, stiff, and misshapen elbow^1,8^.

The proximal radioulnar joint's location to the distal humerus determines the classification of elbow dislocations: posterior, anterior, medial, or lateral. There are two types of posterior dislocations: posterolateral and posteromedial. 90% of elbow dislocations occur on the posterolateral side and are brought on by falls onto the outstretched hand with the forearm pronated^1,9^.

Simple dislocations are defined as dislocations without accompanying fractures. Complex dislocations are those that result from fractures of the olecranon, distal humerus, radial head or neck, or coronoid process. Brachial artery, median nerve, and ulnar nerve damage are examples of neurovascular injuries^1,3,10^.

According to Martini *et al*, every patient should have surgery if their pre-operative elbow flexion was less than 80°^11^. When the arm can only flex to a maximum of 80° while compensating with the shoulder and hand, the stiffness renders the arm useless^2,4,12^.

Surgery is simpler if the dislocation occurred less than six months ago since there is less tissue (ligament and triceps) retraction. The case for surgery is not as clear-cut when the dislocation is older (older than six months). Our experience has shown that the dislocation should be surgically minimised if it causes the patient to have less than 90° of elbow flexion. Because food is eaten by hand in our location and the right arm is the sole one used for this purpose, even patients with 90° elbow flexion are unable to reach their hand to their mouth^2,4,12^.

For open reduction, the posterior and lateral methods are most commonly used. The first report of surgical therapy for neglected elbow dislocation came from Speed, who used a posterior route that allows for adequate relaxation of all constricted tissues and optimum exposure. This method has been applied for many years all over the world. Its effectiveness, however, has been noted to decrease if applied in situations involving more than three months of chronicity due to widespread soft tissue fibrosis, articular cartilage degradation, and localised osteoporosis^2,4^.

We favoured the posterior approach because, in cases of neglected elbow dislocation in low- and middle-income country settings, this approach has shown promising outcomes^13,14^. It offers excellent surgical exposure, minimises soft tissue disruption, and reduces the risk of nerve injury. Additionally, the posterior approach allows the surgeon to address both the dislocation and its associated complications, such as soft tissue contractures that commonly develop due to prolonged immobilisation^13,14^. In this study, the posterior approach facilitated joint stabilisation through capsular release and ligament repair, with minimal damage to the surrounding soft tissues and enabled ulnar nerve transposition. Thus, this method provides optimal access to the joint and contracted soft tissues, allowing for direct visualisation during joint manipulation and reduction. It also offers good exposure to the posterior components that are normally retracted. Direct visual control reduces and fixes the joint. Since there is only one surgical scar, the overall appearance is also more desirable^5,15^.

All contracted soft tissues be released adequately by circumferential subperiosteal stripping from the distal humerus, followed by scar tissue and callus excision. The periosteum is sutured back together after the reduction. The author also did not perform boxloop and pinning because it can cause joint damage and stiffness^16^. This can be done on the condition that all soft tissue is reattached to its place. Patient with onset more than three months, posterior approach is more recommended than the double approach, this is likely to produce suboptimal results but is still worthwhile to gain long term functional improvement and have better result. On the other hand, this procedure may have risk to create extensive soft tissue fibrosis, articular cartilage degeneration and regional osteoporosis^15,17^.

In this study, the sample obtained was dominated by men with a male-to-female ratio is 1.8:1. This is in line with several existing studies, where the prevalence of men compared to women is higher at around 1.23–3.4:1.2–4 Regarding the age of the research sample, it was found that the average age was 31 years, which is in the adult category. This is also by existing research with results between 28–36 years^2-4^.

The MEPI score and Quick DASH questionnaire are used to assess post-operative elbow joint function because both tools are easy to use, accommodate both clinical evaluation by physicians and patient-reported outcomes, and are valid and reliable for studies related to upper limb function^18,19^. Quick DASH (Disabilities of Arm, Shoulder, and Hand) scoring is easier to use and interchangeable with standard DASH scoring. Both the MEPI and DASH scores range from 0 to 100; however, the DASH score has a worst outcome of 100, and the MEPI score is equal to the best result. Other than the fact that a score of zero denotes no disability and a score of 100 denotes entire disability, there are no set guidelines for what constitutes a very excellent, good, satisfactory, or bad score in the DASH system. So, we use the DASH grading reference from existing research with the interpretations of excellent (0–5), good (6–15), fair (16–35), and poor (>36)6.

The elbow function was evaluated pre-operatively, postoperatively, and at every follow-up using the Mayo Elbow Performance Index. There are four parameters in this rating system: Elbows that are pain-free score 45 points, while elbows that are pain-free yet allow for normal elbow movement score 20, stable elbows score 10, and five everyday activities score 25 points. Results are ranked as very good (90–100), good (75–89), fair (60–74), or bad (<60) based on Mayo's score^3,20^.

The research results regarding the functional outcomes of the research samples treated with the posterior approach surgical technique are quite good. The patient's average DASH score was 15 which has a good interpretation. In research by Prasetia et al, two cases of neglected elbow dislocation were presented which had lower DASH score than our study, namely 32.5 and 29.2, this concludes in fair category. Also, there is research that is almost the same as ours which has lower results, namely a mean score of 36 with poor interpretation. This finding may also be biased due to the smaller sample size in that study compared to ours, as well as the existence of two different approaches within the technique, which may have contributed to the lower value^2,21^.

The next patient outcome results with the MEPI score obtained an average of 90 which is in the excellent category. This is more superior than Sumarwoto's research which carried out two approaches and with several studies using only the posterior approach, namely at numbers 86.7 and 85.62,21.

This study also demonstrated the correlation of several variables with patient outcomes. Starting from gender, it was found that men had better outcomes based on the evaluation of DASH and MEPI scores. The results of the study are still not well representative and there is bias due to the non-homogeneous sample size between men and women. In the age variable, the results show that if the sample age is younger, the DASH score will be lower and the MEPI score will be higher. This means that the results at a young age are better. This is in line with the research of Anderson et al which also used a posterior approach. In the case of the dual approach, something similar was also found^2,10,21^.

Research subjects were also given data regarding the duration between the first symptoms felt and surgery. Even though the results are not statistically significant, it can be shown that the shorter the duration, the better the results. In line with existing theory, the most optimal results are an onset of less than three months. After that, extensive soft tissue fibrosis, articular cartilage degeneration, and regional osteoporosis will occur. Even though the start is >3 months, surgery can still be carried out, but usually, the results are less than optimal. On the other hand, some sources say that the results do not affect the starting point, everyone can have a good outcome if there is no significant retraction and the triceps can be mobilised^1,4^.

## CONCLUSION

Neglected dislocations are a fact of life in our country. Patients with neglected elbow dislocations are mostly men and adults. Although surgical therapy is difficult, the improvement and recovery of function is dramatic. There are many benefits to using a posterior approach. The results of the posterior surgical approach provide quite good results in the DASH and MEPI scores. The literature on this topic is limited, and more research is needed to reach consensus.

## CONFLICT OF INTEREST

The authors declare no potential conflict of interest.
